# Frequency of Participation in External Quality Assessment Programs Focused on Rare Diseases: Belgian Guidelines for Human Genetics Centers

**DOI:** 10.2196/27980

**Published:** 2021-07-12

**Authors:** Joséphine Lantoine, Anne Brysse, Vinciane Dideberg, Kathleen Claes, Sofie Symoens, Wim Coucke, Valérie Benoit, Sonia Rombout, Martine De Rycke, Sara Seneca, Lut Van Laer, Wim Wuyts, Anniek Corveleyn, Kris Van Den Bogaert, Catherine Rydlewski, Françoise Wilkin, Marie Ravoet, Elodie Fastré, Arnaud Capron, Nathalie Monique Vandevelde

**Affiliations:** 1 Rare Diseases Unit Department of Quality of Laboratories Sciensano Brussels Belgium; 2 Center of Human Genetics CHU of Liège University of Liège Liège Belgium; 3 Center for Medical Genetics Ghent University Hospital Gent Belgium; 4 Department of Quality of Laboratories Sciensano Brussels Belgium; 5 Center of Human Genetics Institut de Pathologie et de Génétique Gosselies Belgium; 6 Center for Medical Genetics Universitair Ziekenhuis Brussel Vrije Universiteit Brussel Brussels Belgium; 7 Center of Medical Genetics Antwerp University Hospital and University of Antwerp Edegem Belgium; 8 Center for Human Genetics University Hospitals Leuven Leuven Belgium; 9 Center of Human Genetics Hôpital Erasme Université Libre de Bruxelles Brussels Belgium; 10 Center for Human Genetics Cliniques universitaires Saint-Luc Université catholique de Louvain Brussels Belgium

**Keywords:** human genetics, external quality assessment, quality control, proficiency testing, frequency, genetic testing, rare diseases, cost-effectiveness, surveillance, public health authorities, public health, health informatics, medical informatics, genetics, human genetics, algorithm

## Abstract

**Background:**

Participation in quality controls, also called external quality assessment (EQA) schemes, is required for the ISO15189 accreditation of the Medical Centers of Human Genetics. However, directives on the minimal frequency of participation in genetic quality control schemes are lacking or too heterogeneous, with a possible impact on health care quality.

**Objective:**

The aim of this project is to develop Belgian guidelines on the frequency of participation in quality controls for genetic testing in the context of rare diseases.

**Methods:**

A group of experts analyzed 90 EQA schemes offered by accredited providers and focused on analyses used for the diagnosis of rare diseases. On that basis, the experts developed practical recommendations about the minimal frequencies of participation of the Medical Centers of Human Genetics in quality controls and how to deal with poor performances and change management. These guidelines were submitted to the Belgian Accreditation Body and then reviewed and approved by the Belgian College of Human Genetics and Rare Diseases and by the National Institute for Health and Disability Insurance.

**Results:**

The guidelines offer a decisional algorithm for the minimal frequency of participation in human genetics EQA schemes. This algorithm has been developed taking into account the scopes of the EQA schemes, the levels of experience, and the annual volumes of the Centers of Human Genetics in the performance of the tests considered. They include three key principles: (1) the recommended annual assessment of all genetic techniques and technological platforms, if possible through EQAs covering the technique, genotyping, and clinical interpretation; (2) the triennial assessment of the genotyping and interpretation of specific germline mutations and pharmacogenomics analyses; and (3) the documentation of actions undertaken in the case of poor performances and the participation to quality control the following year. The use of a Bayesian statistical model has been proposed to help the Centers of Human Genetics to determine the theoretical number of tests that should be annually performed to achieve a certain threshold of performance (eg, a maximal error rate of 1%). Besides, the guidelines insist on the role and responsibility of the national public health authorities in the follow-up of the quality of analyses performed by the Medical Centers of Human Genetics and in demonstrating the cost-effectiveness and rationalization of participation frequency in these quality controls.

**Conclusions:**

These guidelines have been developed based on the analysis of a large panel of EQA schemes and data collected from the Belgian Medical Centers of Human Genetics. They are applicable to other countries and will facilitate and improve the quality management and financing systems of the Medical Centers of Human Genetics.

## Introduction

Rare diseases are life-threatening or chronically debilitating conditions affecting less than 5 per 10,000 people [1]. At least 80% of them have a genetic origin and 50% to 75% affect children [2,3]. Despite the discovery of more than 200 new genes every year, the diagnosis of rare diseases often remains delayed because of their complexity and low prevalence [1,4-6].

**Figure 1:**
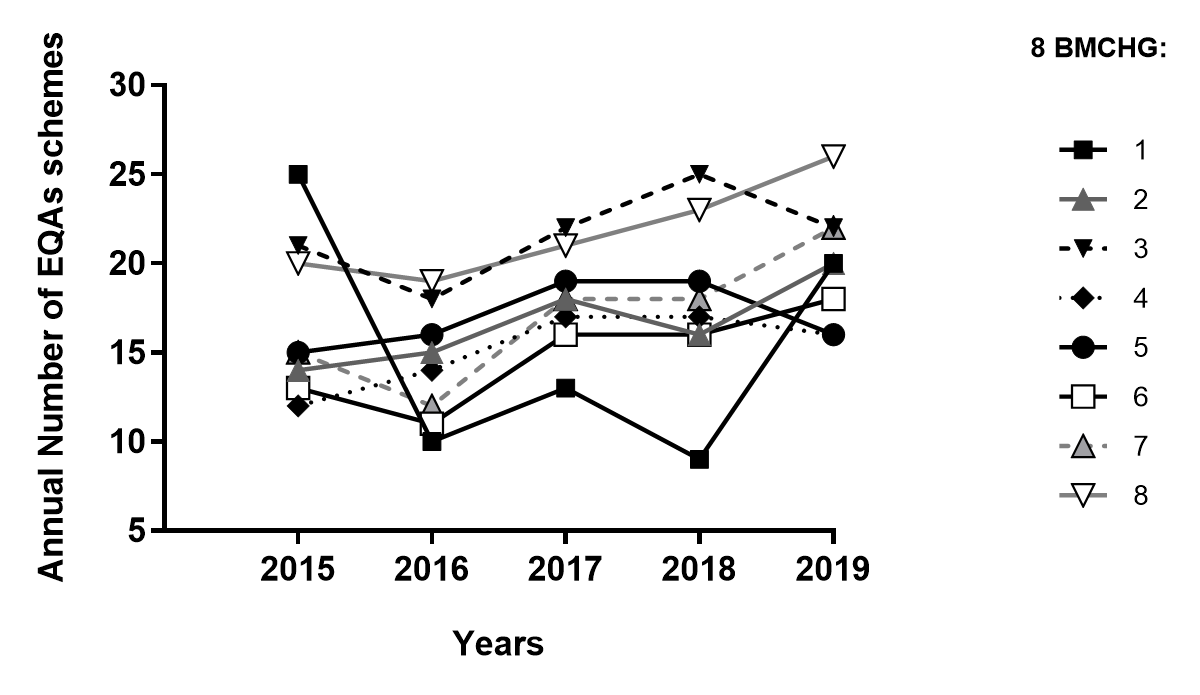
Evolution of the participation of the 8 BMCHG to the inventoried EQA schemes between 2015 and 2019. BMCHG: Belgian Medical Centers of Human Genetics; EQA: external quality assessment.

There is a willingness of European governments to develop harmonized guidelines to improve the quality of genetic testing, particularly by stimulating medical laboratories to acquire accreditation and to participate in external quality assessments (EQAs) [7,8]. Indeed, the participation in EQA schemes is efficient for assessing and improving health care quality, as it allows performance comparisons and the identification of specific problems, areas for improvement, and training needs [9-11]. Besides, it enables monitoring of the compliance with best practice guidelines and is required for the accreditation of medical laboratories according to the ISO 15189 standard [12-14]. It has been shown that the quality of genetic services in Europe can still improve [10,15]. Nevertheless, several recent studies performed in European laboratories for cancer testing have pointed out the positive influence of participation in EQAs on laboratories’ performance [16-18]. In several countries such as in Belgium, the accreditation of the genetic laboratories is a requisite for reimbursement of the diagnostic tests. However, the EQA of the laboratories is still hampered by a lack of a harmonized European framework (numerous and heterogeneous quality schemes, lack of reference systems, and different Member State regulations) [19,20]. Similar concerns have been raised in a recent Belgian study focusing on the frequency of participation in EQA schemes in the fields of molecular microbiology, hematology, and pathology [20]. The authors proposed to harmonize the frequency of participation to quality controls [20]. Indeed, the ISO 15189 standard states that “the laboratory shall participate in an inter-laboratory comparison program (such as an EQA program or proficiency testing program) appropriate to the examination and interpretations of examination results,” but does not give precise instructions [13]. This lack of clear national and international directives leads to uneven participation of the Medical Centers of Human Genetics in quality controls [21]. Figure 1 illustrates this phenomenon with the participation of the Belgian Medical Centers of Human Genetics (BMCHG) in EQAs between 2015 and 2019.

To address this lack, we have developed Belgian guidelines about the minimal frequency of participation in EQA schemes for hereditary rare diseases, with reference to international recommendations and national laboratory practices.

## Methods

### Context of the Study

In the context of the Belgian National Plan for Rare Diseases, the Belgian National Institute for Health (Sciensano [22]) is responsible for the harmonization of the quality management system for rare disease diagnostics within the BMCHG.

### Data Collection

In 2018, Sciensano performed a preliminary inventory of 90 EQA schemes related to rare diseases and that the BMCHG participate in. Of note, in the case of cancers, only EQA schemes for rare hereditary cancers were considered, while schemes for somatic mutation detection were excluded. In 2019, Sciensano collected retrospective data about the annual participation of the BMCHG in the inventoried EQA schemes between 2015 and 2019.

### Guidelines for the Participation to Genetic EQA Schemes

To structure and harmonize the frequency of participation of the BMCHG in EQA schemes focused on the genetic diagnosis of rare diseases, a working group composed of two representatives for each of the 8 BMCHG was established by Sciensano in 2019, in consultation with the Belgian College of Human Genetics and Rare Diseases [23].

The working group developed recommendations about the minimal frequency of participation of the BMCHG in quality controls. These recommendations were accompanied by a decisional algorithm to help the BMCHG to plan their future participations in quality controls based on their own experience in the performance of the tests considered and the scopes of the available EQA schemes. Besides, attention was paid to recommendations on actions that should be undertaken in case of poor performance to EQA schemes and to the continuous follow-up and surveillance of the participation of the BMCHG to EQA schemes.

### Validation of the Guidelines

The inventory of 90 genetic EQA schemes focused on rare diseases was used by the working group for the validation of the decisional algorithm based on the routine BMCHG practice.

Besides, the opinion of three accreditation managers from the Belgian Accreditation Body regarding the whole guidelines’ draft was requested. The final version of the guidelines was submitted in 2020 to the Belgian College of Human Genetics and Rare Diseases for evaluation and endorsement.

### Study Workflow

**Figure 2:**
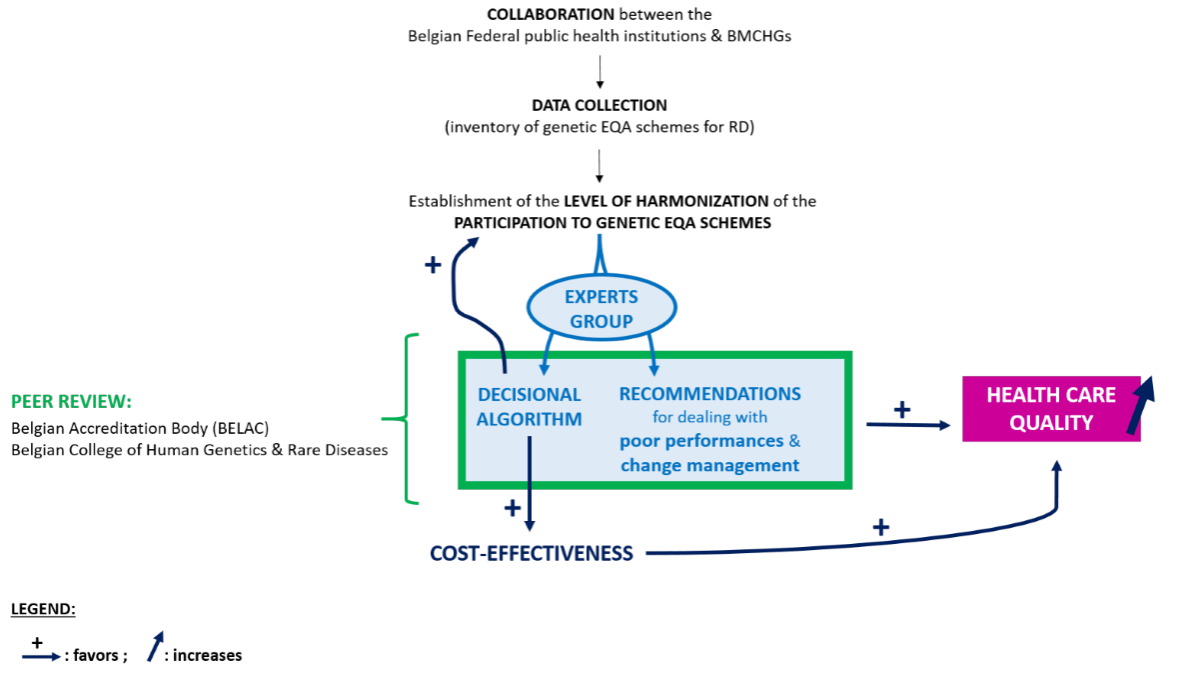
Workflow and achievements of the study. BMCHG: Belgian Medical Centers of Human Genetics; EQA: external quality assessment; RD: rare diseases.

The global study workflow is illustrated in Figure 2.

### Statistical Analysis

For some rare disorders, only a few requests are obtained on an annual basis. Due to this low number, the question now arises whether the number of routine analyses can be used as an indicator of performance. Indeed, in genetic testing, errors have a great impact on the patients’ and their relatives’ lives. It is therefore important to maintain a high quality level, even if every laboratory is subject to underlying errors. To this aim, an error rate of 1% has been set by the working group as the maximal error threshold to define the quality of the performance of genetic tests. This threshold has been determined based on the following:

- The error rate reported in May 2020 by the EQA provider European Molecular Quality Network [24] for its global data for the germline schemes organized by this provider during the past 5 years (2016-2020); mean analytical error rate 1.37% (unpublished data): This percentage is based on the assessment of more than 33,132 genotypes during 11,044 participations in fully operational EQA schemes for germline mutation testing (technical, molecular pathology, and pilot schemes were excluded; each scheme assesses 3-4 samples). Of note, the mean analytical error rate is defined as any genotyping error that would lead to patient harm.
- Data published in the scientific literature: Indeed, error rates between 0.1% and 1% have been reported for high-throughput DNA sequencing technologies (eg, next generation sequencing) [25,26]. Raw data about error rate percentages published in 5 other peer-reviewed scientific papers for different types of situations (diseases, techniques, etc) [27-31] were also analyzed. Mean error rates and SDs with the number of scenarios investigated by the authors and confidence intervals are reported in Table 1. Based on this analysis it appears that the mean error rates fluctuate approximately between 0% and 4%.

**Table 1:** Data analysis about error rate percentages published in peer-reviewed papers for different types of situations.

Bibliographic references	Investigated scenarios, n^a^	Error rate (%), mean (SD)	95% CIs of the mean (%)
Hofgartner et al, 1999 [27]	8	0.38 (0.34)	0.10 to 0.67
Ewen et al, 2000 [28]	7	0.89 (0.82)	0.13 to 1.65
Bonin et al, 2004 [29]	4	2.20 (1.10)	0.46 to 3.94
Hoffman and Amos, 2005 [30]	8	0.35 (0.19)	0.19 to 0.50
Gilles et al, 2011 [31]	2	0.80 (0.38)	–2.63 to 4.23

^a^Number of investigated scenarios for different types of situations reported in the literature.

To determine the sufficient number of analyses needed to have a maximal error rate of 1%, assuming that the laboratory is performing well, the distribution of possible error rates for a certain performance statistic was modeled using the *proportion* library of R software (version 3.6.1; R Foundation for Statistical Computing).

A Bayesian model with noninformative prior was used for having a rate of 100% correct analyses for a certain number of analyses and a rate of (n – 1) / n correct analyses for a certain number of analyses n. Details concerning the statistical model can be found in Multimedia Appendix 1.

## Results

### Scope of Guidelines

As this study was funded by a grant dedicated to the improvement of the quality of the genetic testing in the BMCHG in the context of rare diseases, all developed guidelines are related to human genetics EQA schemes for rare hereditary diseases, including germline predispositions to cancers and adverse drug effects resulting from pharmacogenomic variants [32].

### EQA Schemes Inventory

The EQA schemes inventoried during the preliminary phase of the study and considered by the working group for the establishment of the guidelines are mentioned in Multimedia Appendix 2 [24,33-38]. They are focused on the diagnosis of 72 different rare diseases or specific genetic variants involved in rare diseases. For each scheme, we have reported the aspects that are assessed (technique, genotyping, and interpretation of the results). The majority (n=65, 72%) of the EQA schemes are assessing the technique, genotyping, and interpretation. A total of 21 (23%) of the schemes are assessing both the technique and genotyping. A few of the schemes are covering both the genotyping and interpretation (n=1, 1%), only the technique (n=1, 1%), only the genotyping (n=1, 1%), or only the interpretation (n= 1, 1%). Of note, for 15 selected rare diseases or genetic variants, EQA schemes are offered by 2 or 3 providers, which increases the total number of inventoried EQA schemes (n=90) used for the percentage calculations described here. These EQA schemes are marked with an asterisk in Multimedia Appendix 2.

### Guidelines

**Figure 3:**
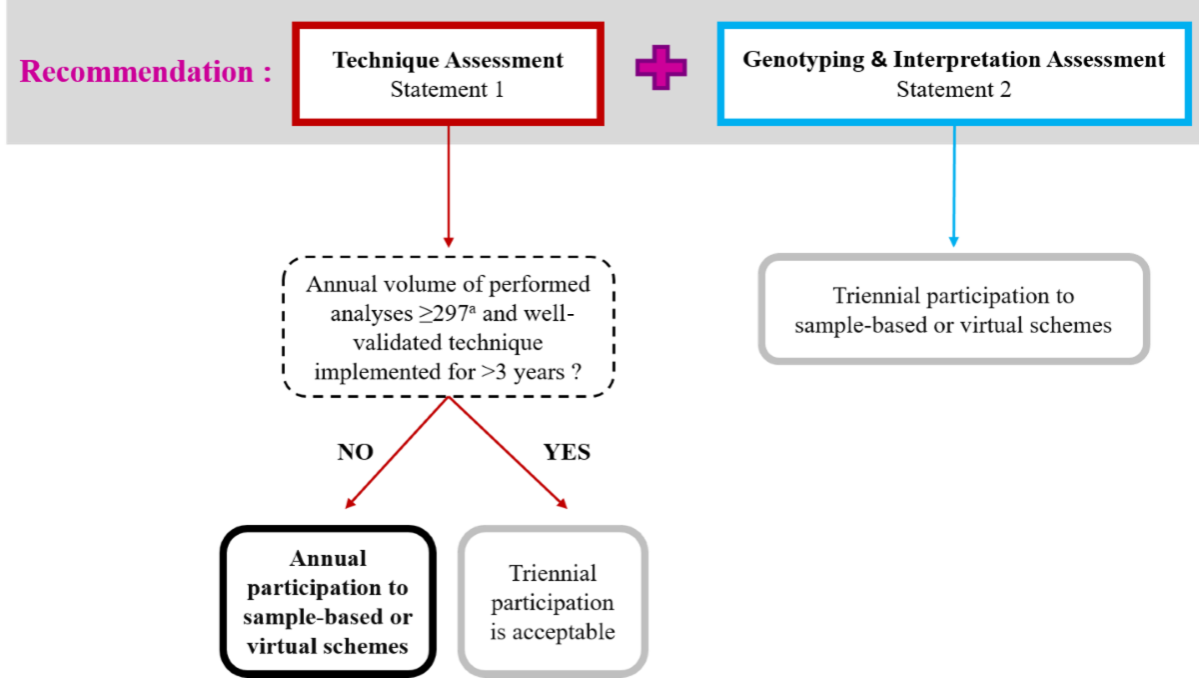
Decisional algorithm for the minimal frequency of participation to external quality assessment schemes (cf to statements 1 and 2 of the Results). ^a^Number of analyses required to have a maximal error rate ≤1%.

These guidelines on the minimal frequency of participation in EQA schemes can be divided into three pillars: (1) general recommendations that constitute the backbone of the guidelines and cover the majority of situations encountered (statements 1-2 and Figure 3), (2) how to address poor performances (statements 3-4), and (4) follow-up and surveillance (statements 5-6).

#### General Recommendations

The quality of all diagnostic tests offered by the BMCHG should be frequently assessed. Indeed, the annual rate of some specific analyses (eg, performed in the context of rare diseases) may be very low. However, given the large number of genetic diseases and the low frequency of most of them, an annual participation in all possible EQA schemes is neither feasible nor economically defendable. It is therefore important to run a quality assessment in a comprehensive, rather than an exhaustive way, to assess the quality of services offered to the patients. We also took into account that the same techniques are applied for analyses of different rare disorders.

##### Statement 1: Annual Assessment of All Techniques

The quality of all techniques and technological platforms used by the BMCHG should be annually assessed, even with EQA schemes only based on mock clinical cases, virtual images or variant call format files, or raw data sets. As mentioned by Brookman et al [39], if visual inspections are needed in daily practice, virtual schemes are useful to test postanalytical performances, notably in the case of fluorescence in-situ hybridization and karyotype analysis.

If different EQA schemes exist to cover the same technique, the centers are free to implement a turnover between those EQAs as long as the technique is covered every year and the clinical indications at least every 3 years (cf statement 2).

An exception is made for well-validated methods implemented for more than 3 years (*in-house* or using a commercial CE [European Conformity]–labelled kit) and for which at least 297 tests per year are performed, meaning that the maximal error rate of the analysis is between 0% and 1%. If those specific conditions are met, a triennial participation to an EQA is sufficient to evaluate a specific technique, as long as the methodology does not change. Of note, the number of tests have been deducted from the Bayesian statistical model performed for the distribution of the maximal error rates (see Multimedia Appendix 1). The rationale for triennial participation has to be properly documented in the center’s quality management system [40].

##### Statement 2: Genotyping and Interpretation Assessment

A triennial assessment of the genotyping and interpretation for the detection of specific germline mutation diseases and pharmacogenomics is considered sufficient as long as the technique involved is covered (cf statement 1). This is also true when EQA schemes only assess the clinical interpretation based on virtual clinical cases or images.

#### How to Address Poor Performances

##### Statement 3: Identification of Errors’ Origins

Poor EQA performances because of analytical or clerical errors (eg, copy and paste mistakes) have to be discussed internally. All actions (cause analysis, corrective, and preventive actions) carried out in response to the poor evaluation must be properly documented according to the center’s quality management system procedures.

##### Statement 4: Poor Performances With an Impact on the Diagnosis

In case of poor EQA performance due to genotyping or critical interpretation errors that impacts the diagnosis, the center has to participate in an EQA the following year. Actions taken to avoid future errors have to be documented in the quality management system of the centers.

#### Follow-up and Surveillance

##### Statement 5: Management of Changes in Activities and EQA Schemes’ Availability

It is the responsibility of the Medical Centers of Human Genetics to regularly review and adapt their participation to EQA schemes based on the present guidelines, changes in activities or infrastructure (eg, significant changes in the annual volume of tests and gene panels or modifications in the technique or analytical equipment), and new schemes introduced on the market. This should be notified in their quality management system.

##### Statement 6: Implication of Public Health Authorities

Public health authorities can play a key role in the improvement and follow-up of the activities, quality, and cost-effectiveness of medical laboratories such as the Medical Centers of Human Genetics. For instance, the Belgian National Institute for Health, called Sciensano, will annually coordinate the participation of the BMCHG to EQA schemes focused on rare diseases and hereditary cancers, ensure the reimbursement of participation fees, and monitor the outcomes. To provide this service, the data regarding the participation of BMCHG will be used to forecast the annual global budget dedicated to the reimbursement of participation fees. This information will then be communicated to the Belgian health care authorities. Besides, Sciensano and the working group will also regularly review and update the Belgian guidelines according to the evolution of the centers’ activities, scientific developments, and EQAs’ availability.

In the coming years, the collected data about the participation frequencies of the BMCHG in EQA schemes will be included into the Belgian genetic tests database, developed by Sciensano, in collaboration with the BMCHG.

### Impact of the Guidelines on Health Care Costs

We have studied the impact that the establishment of harmonized guidelines on the minimal frequency of participation of the BMCHG in EQA schemes may have on national health care and genetic centers’ expenditures. To this aim, three different scenarios have been compared:

1. The cost estimation if the BMCHG would annually participate to all EQAs included in their assessment scope among the inventoried EQA schemes focused on 72 rare diseases or genetic variants (fictitious scenario)
2. The participation costs of the BMCHG to the same EQAs as in 2019 (in absence of guidelines)
3. The prediction of the annual BMCHGs participation costs (mean over 2020, 2021, and 2022) for the EQAs included in their assessment scope, following the participation frequencies proposed in the guidelines

Based on the costs of the different EQA schemes, the estimated annual expenditures in these three scenarios were €117,400 (~US $140,444), €82,000 (~US $98,096) and €70,600 (~US $84,458), respectively.

These estimations show that the rationalization of the frequency of participation proposed in these guidelines (third scenario), based on the types of EQA schemes and results of previous participation, enables a reduction in global annual participation costs of 14% for the 8 BMCHG.

Based on the developed guidelines on the minimal frequency of participation and current commercial EQA prices, we were able to estimate that a mean annual budget of €9000 (~US $10,900) is required for each BMCHG to cover the fees requested by the provider to participate in the EQA schemes included in their assessment scope.

## Discussion

### Principal Results and Strengths

A regular participation in quality controls is mandatory for the accreditation of medical laboratories under the ISO 15189 standard [12,14,40]. Accreditation itself is a requisite for the reimbursement of genetic tests in Belgium. However, no Belgian instructions on the required frequency of participation in rare diseases diagnostic and genetic testing EQAs were available prior to this study. This study can be considered as the first Belgian harmonized quality update in terms of frequency of participation in proficiency testing in the field of human genetics.

These guidelines present six main strengths. First, they are based on European recommendations [41-43] and on the clinical and laboratory practice to make them as broad and consistent as possible. Second, they have been developed by a working group composed of representatives of all BMCHG to ensure a harmonization at the national level. Besides, these members have different professional backgrounds and tasks that enabled us to collect the opinions of all stakeholders involved in the performance of different types of genetic tests (molecular, cytogenetic, and biochemical), quality management, and in the interaction with the Belgian health care authorities. Third, a distinction was made based on the aspects assessed by the EQA schemes (technique, analysis, or interpretation) to draft guidelines as relevant as possible. Fourth, a large number of available genetic EQA schemes from accredited providers has been considered. This emphasizes the importance of assessing the quality of highly specific tests performed at a relatively low annual volume in the context of rare diseases. Fifth, a statistical model was used to estimate the probability of a laboratory to make a mistake according to the number of analyses that are performed per year. This new model may help other laboratories to define the minimal number of analyses required to indicate that the experience of a laboratory can be taken into account as a reliable performance indicator. Finally, the guidelines have been approved by the Belgian College of Human Genetics and Rare Diseases and are in accordance with the statements of the ISO 15189 standard referring to the validation of analytical methods [13]. This ensures their clinical relevance and legal accreditation aspects.

Participating in a large number of different EQAs for rare diseases is worthwhile, as it has a role in controlling performance and guarantees permanent education. Furthermore, participating in international EQA schemes enables the performances of a large number of the Centers of Human Genetics to be compared and evaluated by a wide range of international experts. However, taking part in a large number of EQAs is a lot of work and time-consuming. Hence, a balance had to be sought between usefulness and burden. These new Belgian guidelines will improve the harmonization and structuring of the BMCHG quality management system and help the laboratories to identify the EQA schemes that they should participate in based on the evolution of their activities and type of EQA schemes considered. They might also serve as basis for the Belgian Accreditation Body for accreditation assessments and for the Belgian health care authorities to estimate the necessary budget that should be foreseen and attributed by the National Institute for Health and Disability Insurance to the BMCHG to cover participation fees.

### Comparison With Prior Work

Similar recommendations have already been developed by other countries, for instance, Dutch, Slovenian, and Estonian laboratories have to participate in a minimum of one EQA scheme for each accredited analysis of their scope during an accreditation cycle (during 3 years, till the suspension of the accreditation, and during 5 years, respectively), while other (eg, Lithuanian) laboratories are requested to participate twice during this period of time or every year for specific fields [43]. It is unfortunate that no European consensus exists at this time [19]. However, we hope that the development of guidelines on this topic in different European countries should be a catalyst to the initiation of a general reflection on the harmonization of the quality assessment of genetic testing at a European level.

Our guidelines reflect the opinion that the scope of quality controls should be broad enough to cover all methods, technologies, and tests included in the scope of the centers. It is not acceptable that a laboratory would only be accredited for a (small) fraction of its testing offers and thus avoid EQA participation.

### Limitations

Regarding the limits of this study, we have to mention that these guidelines only concern EQA schemes from accredited providers. Ring tests [44] to which BMCHG may also participate in with a small number of other Belgian or foreign genetic centers were excluded. Nonetheless, the preliminary phase of the study revealed that approximately 30% of the quality controls to which the BMCHG participate in are ring tests. They were not considered in this study because we wanted to give priority to EQAs offered by accredited providers. Ring tests are often highly specific and involve a limited number of participants. The difficulty to get enough test material for all participants make the standardization of their organization difficult. However, this opens the door to future improvements in the harmonization process of the quality management of human genetic analyses when no formal EQA scheme is available.

Another limitation is that the majority of the EQAs considered are specific for hereditary rare diseases and not for all diseases.

Finally, the guidelines have been developed at the Belgian level, without asking the opinions of foreign experts. However, several members of the working group act as assessors in international schemes and have good insights into practice, evaluation, and (poor) performance management.

### Conclusion

These first Belgian guidelines will help the BMCHG to improve their quality management system with recommendation on the frequency of participation in EQA schemes and on dealing with poor performance and change management. Moreover, they help the Belgian health care authorities to estimate the budget required to cover the participation of the BMCHG in EQAs. We are convinced that these Belgian guidelines could be used by foreign human genetics medical centers and can serve as a starting point for discussion about the harmonization of quality processes at a broader level.

## Appendix

Multimedia Appendix 1 Statistical modeling of the maximal error rates when performing genetic analyses for rare diseases.

Multimedia Appendix 2 List of the inventoried external quality assessment schemes considered for the establishment of the guidelines. EQA schemes offered by several providers are marked with an asterisk.

## Abbreviations

BMCHG: Belgian Medical Centers of Human Genetics
CE: European Conformity
EQA: external quality assessment
