# Supplementary material for: Frequency of Participation in External Quality Assessment Programs Focused on Rare Diseases: Belgian Guidelines for Human Genetics Centers
Source: JMIR Med Inform. 2021 Jul 12;9(7):e27980. doi: 10.2196/27980 (PMC8314149; doi:10.2196/27980)
Supplement: Multimedia Appendix 1 [file medinform_v9i7e27980_app1.docx]

**Description of the statistical analysis performed to determine the maximal error rates corresponding to a certain number of performed analyses.**

Some analyses used for the diagnosis of rare diseases are performed only a few times per year in the Belgian medical centers of human genetics (BMCHGs) due to the low number of requests.

The question now arises whether the number of routine analyses can be used as an indicator of the performance and be implemented as a criterion to define the frequency of participation to External Quality Assessment Schemes. As it is difficult to provide a consistent number of analyses, a statistical model was used in our study to calculate the maximal error rates for a certain number of correct analyses.

Indeed, some may argue that laboratories that do not perform a specific test often, will perform better because their uncertainty will make them to pay more attention to the analysis. Others may argue that a certain experience is necessary in order to reliably perform a test requiring then, frequent testing. The problem with these arguments is that they are difficult to quantify.

In this study, several assumptions were taken into account for the statistical model:

• All the data are independent meaning that the probability to make a mistake for the analysis X is not influenced by the result of analysis X-1. Since we haven’t the knowledge to make a correlation between the analyzes X and X-1, we presume that this correlation is zero.

• The statistical model is based on a Bayesian informative prior model assuming that we have no idea of the real error rate of the BMCHGs and then we cannot make any pre-supposition about it.

The result of the statistical modeling are presented in Table S1. The distribution of possible error rates for a certain performance statistic was modeled according to this Bayesian model for (i) having a rate of 100% correct analyses and (ii) having a rate of (n-1)/n correct analyses for a certain number of analyses n.

For several number of analyses ranging from 1 to 1000, the one-sided 95% upper confidence interval of the distribution of possible proportions was calculated.

The results presented in Table S1 should be interpreted as follows: imagine that n or (n-1)/n results are correct, what could be the maximum underlying error rate?

The results are given each time on a significance level of 5%, meaning that there is a probability of 5% of having a higher number of error rates.

**Table S1. Distribution of the maximal error rate according to the number of routine analyses (n).**

| **Number of analyses (n) performed with the technique per year** | **Maximum probability (%) of making a mistake if all results are correct, 95% confidence** | **Maximum probability (%) of making a mistake if one result is wrong among the n analyses** |
| --- | --- | --- |
|  |  |  |
| - 1 | 77.64% | 100% |
| - 2 | 63.16% | 86.46% |
| - 3 | 52.71% | 75.14% |
| - 4 | 45.07% | 65.74% |
| - 5 | 39.30% | 58.18% |
| - 10 | 23.84% | 36.44% |
| - 15 | 17.07% | 26.40% |
| - 20 | 13.29% | 20.67% |
| - 25 | 10.88% | 16.98% |
| - 30 | 9.21% | 14.41% |
| - 35 | 7.98% | 12.51% |
| - 40 | 7.05% | 11.06% |
| - 45 | 6.30% | 9.90% |
| - 50 | 5.70% | 8.97% |
| - 55 | 5.21% | 8.19% |
| - 60 | 4.79% | 7.54% |
| - 65 | 4.44% | 6.99% |
| - 70 | 4.13% | 6.51% |
| - 75 | 3.87% | 6.09% |
| - 80 | 3.63% | 5.72% |
| - 85 | 3.42% | 5.40% |
| - 90 | 3.24% | 5.11% |
| - 95 | 3.07% | 4.85% |
| - 100 | 2.92% | 4.61% |
| - 200 | 1.48% | 2.34% |
| - **297^a^** | **1.00%** | **1.58%** |
| - 300 | 0.99% | 1.57% |
| - 400 | 0.74% | 1.18% |
| - 500 | 0.60% | 0.94% |
| - 600 | 0.50% | 0.79% |
| - 700 | 0.43% | 0.67% |
| - 800 | 0.37% | 0.59% |
| - 900 | 0.33% | 0.53% |
| - 1000 | 0.30% | 0.47% |
| **^a^A maximal error rate of 1 % and so the performance of ≥297** **tests per year** is the threshold chosen to bypass the annual assessment of a well-validated technique implemented for more than 3 years. In that case, a triennial participation to an EQA is sufficient in order to evaluate the specific technique. | | |
