# Supplementary material for: Frequency of Participation in External Quality Assessment Programs Focused on Rare Diseases: Belgian Guidelines for Human Genetics Centers
Source: JMIR Med Inform. 2021 Jul 12;9(7):e27980. doi: 10.2196/27980 (PMC8314149; doi:10.2196/27980)
Supplement: Multimedia Appendix 2 [file medinform_v9i7e27980_app2.docx]

**Multimedia Appendix 2.** List of the inventoried external quality assessment schemes considered for the establishment of the guidelines. EQA schemes offered by several providers are marked with an asterisk.

| **EQA Providers** | **EQA schemes** | **Aspects covered by the EQA schemes** | | |
| --- | --- | --- | --- | --- |
|  |  | **Technique** | **Genotyping** | **Interpretation** |
|  |  |  | | |
| **EMQN [24]** |  |  |  |  |
|  | APOB* | X | X | X |
|  | Cardiac Arrhythmias* | X | X | X |
|  | Autosomal Dominant Polycystic Kidney disease | X | X | X |
|  | Beckwith - Wiedemann & Silver-Russell syndromes | X | X | X |
|  | Charcot-Marie-Tooth disease/Hereditary Liability to Pressure Palsies | X | X | X |
|  | Congenital Adrenal Hyperplasia | X | X | X |
|  | Cystic fibrosis* | X | X | X |
|  | DNA Sequencing Sanger | X | X |  |
|  | DNA Sequencing NGS (Germline testing only)* | X | X |  |
|  | Duchene/Becker Muscular Dystrophy | X | X | X |
|  | Familial autosomal dominant hypercholesterolemia | X | X | X |
|  | Familial SHOX-related disorders | X | X | X |
|  | Fragile X Syndrome | X | X | X |
|  | Friedreich Ataxia | X | X | X |
|  | Hereditary Breast and Ovarian Cancer Panel testing version | X | X | X |
|  | Hereditary Deafness | X | X | X |
|  | Hereditary Haemochromatosis* | X | X | X |
|  | Hereditary Recurrent Fevers | X | X | X |
|  | Hereditary retinoblastoma | X | X | X |
|  | Huntington Disease | X | X | X |
|  | Hypertrophic Cardiomyopathies* | X | X | X |
|  | Lynch Syndrome | X | X | X |
|  | Mitochondrial DNA-Metabolic Disorders* | X | X | X |
|  | Monogenic Diabetes | X | X | X |
|  | Multiple Endocrine Neoplasia type 2 | X | X | X |
|  | Myotonic Dystrophy | X | X | X |
|  | Non-invasive prenatal testing for common aneuploidies* | X | X | X |
|  | Osteogenesis imperfecta | X | X | X |
|  | Phenylketonuria | X | X | X |
|  | Polyposis syndromes | X | X | X |
|  | Postnatal Constitutional CNV Detection* | X | X | X |
|  | Prader-Willi and Angelman Syndromes | X | X | X |
|  | RYR1 related myopathies and malignant hyperthermia | X | X | X |
|  | Spinal Muscular Atrophy | X | X | X |
|  | Spinocerebellar Ataxia’s | X | X | X |
|  | Von Hippel Lindau Syndrome* | X | X | X |
|  | Wilson disease | X | X | X |
|  | Y-Chromosome Microdeletions | X | X | X |
| **GenQA [33]** |  |  |  |  |
|  | Ataxia and hereditary spastic paraplegia | X | X | X |
|  | Cardiac Arrhythmias* | X | X | X |
|  | Cardiomyopathies | X | X | X |
|  | Blood – postnatal |  | X | X |
|  | Cystic fibrosis from blood spot | X | X |  |
|  | DNA Sequencing NGS* | X |  |  |
|  | Fabry’s disease | X | X | X |
|  | Familial endocrine tumour predisposition disorders | X | X | X |
|  | Hypertrophic Cardiomyopathies* | X | X | X |
|  | Mitochondrial DNA-Metabolic Disorders* | X | X | X |
|  | Molecular Rapid Aneuploidy QF-PCR | X | X | X |
|  | Non-invasive prenatal testing for common aneuploidies* | X | X | X |
|  | Non-invasive prenatal diagnosis for common microdeletions pilot | X | X | X |
|  | Pathogenicity of sequence variants | X | X | X |
|  | Postnatal Constitutional CNV Detection* | X | X | X |
|  | Preimplantation Genetic Diagnosis of trophectoderm and/or blastomere testing for monogenic disorders | X | X | X |
|  | Preimplantation Genetic Testing of Blastomere/Trophectoderm for chromosomal rearrangement by NGS and/or arrays | X | X | X |
|  | Preimplantation Genetic Testing by NGS and/or Arrays for Trophectoderm and/or Blastomere Testing for Aneuploidies | X | X | X |
|  | Preimplantation Genetic Testing for Blastomere FISH (Stage 1 & 2) | X | X | X |
|  | Prenatal constitutional CNV detection | X | X | X |
|  | Rapid Prenatal Aneuploidy FISH | X | X | X |
|  | RETT syndrome* | X | X | X |
|  | Severe developmental delay |  |  | X |
|  | Skeletal dysplasis | X | X | X |
|  | Von Hippel Lindau Syndrome* | X | X | X |
| **CF Network [34]** |  |  |  |  |
|  | Cystic fibrosis* | X | X | X |
| **CAP [35]** |  |  |  |  |
|  | DPYD* | X | X | X |
|  | Mitochondrial DNA-Metabolic Disorders* | X | X | X |
|  | RETT syndrome* | X | X | X |
|  | TPMT* | X | X | X |
|  | UGT1A1* | X | X | X |
| **UKEQAS [36]** |  |  |  |  |
|  | Alpha and Beta Globin Mutations | X | X | X |
|  | Genetics of Heritable Bleeding & Thrombotic Disorders | X | X | X |
|  | HLA B°5701 | X | X |  |
| **INSTAND [37]** |  |  |  |  |
|  | Alpha1-Antitrypsin | X | X |  |
|  | APOB* | X | X |  |
|  | APOE | X | X |  |
|  | Hereditary Haemochromatosis* | X | X |  |
|  | TPMT* | X | X |  |
|  | VKORC1* | X | X |  |
| **RfB [38]** |  |  |  |  |
|  | ABCB1 | X | X |  |
|  | ALDOB |  | X |  |
|  | CYP2C9 | X | X |  |
|  | CYP2C19 | X | X |  |
|  | CYP2B6 | X | X |  |
|  | CYP2D6 | X | X |  |
|  | CYP3A5 | X | X |  |
|  | DPYD* | X | X |  |
|  | Hereditary Haemochromatosis* | X | X |  |
|  | TPMT* | X | X |  |
|  | UGT1A1* | X | X |  |
|  | VKORC1* | X | X |  |
